# Supplementary material for: Ethnic inequality and forced displacement
Source: PLoS One. 2022 Apr 8;17(4):e0266448. doi: 10.1371/journal.pone.0266448 (PMC8992982; doi:10.1371/journal.pone.0266448)
Supplement: S1 Appendix — (PDF) [file pone.0266448.s001.pdf]

Supporting information

S1 Fig. Horizontal Inequality and Forced Displacement Notes: Correlation of our main variable of interest across communities. (Sources: Life in Kyrgyzstan (LiK) study 2010 [7] and [37].

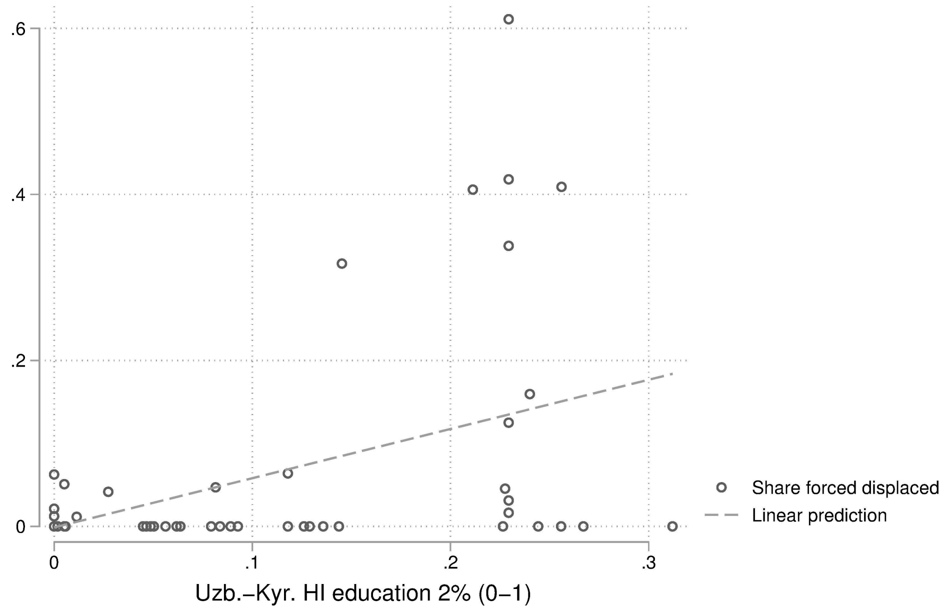

S2 Table. Summary statistics individuals

	Kyrgyz	Uzbeks	Difference	P-Value
Forcefully displaced (No=0,Yes=1)	0.016	0.112	-0.096	0.000
IDP (No=0,Yes=1)	0.016	0.087	-0.071	0.000
Refugee (No=0,Yes=1)	0.000	0.022	-0.022	0.000
Education(completed years)	11.716	10.780	0.936	0.000
Age 18-45 (years)	0.623	0.632	-0.009	0.605
Age 46-99 (years)	0.333	0.325	0.009	0.601
Female (No=0,Yes=1)	0.501	0.509	-0.008	0.665
Has children (No=0,Yes=1)	2.626	2.404	0.222	0.003
Is married (No=0,Yes=1)	0.810	0.816	-0.006	0.647
Father sec edu (No=0,Yes=1)	0.680	0.605	0.075	0.000
Self employed (No=0,Yes=1)	0.197	0.219	-0.022	0.111
Born here (No=0,Yes=1)	0.721	0.926	-0.205	0.000
Land owner (No=0,Yes=1)	0.785	0.761	0.024	0.097
Dist. to Uzb. border	1.44e+05	26792.737	1.17e+05	0.000
Observations	6268			

Note: The sample is based on all individual respondents of the LiK study older than 17 years.

S3 Table. Forced displacement in communities: Outliers

	A<br>b/se	B<br>b/se	C<br>b/se	D<br>b/se
Uzb-Kyr HI education (0-1)	.61***	.43*	.79***	.53*
	(.22)	(.24)	(.27)	(.31)
Gini years of education (0-1)	.26	.094	-.2	-.27
	(.38)	(.27)	(.43)	(.22)
Gini education Uzbeks (0-1)	.2	.24	-.008	.068
	(.29)	(.17)	(.32)	(.086)
Gini education Kyrgyz (0-1)	-.098	-.07	.44	.31
	(.3)	(.21)	(.38)	(.21)
Ethnic polarization (0-1)	-.042	-.025	-.039	-.021
	(.034)	(.029)	(.031)	(.03)
Controls	Yes	Yes	Yes	Yes
Excluded	No	Osh City	Osh	Osh City, Osh
R2	.36	.26	.46	.37
Observations	120	114	101	95

Note: Sample based on 120 sampling communities in LiK study excluding one or two provinces. Coefficients are from OLS regressions. Standard errors are robust. The dependent is the fraction of displaced population from the total population. Horizontal inequality defined in equation (1) with Census 2009 data. Vertical inequality are gini coefficients as defined in section IV.D based on years of completed schooling from the LiK 2010 study. Ethnic polarization as defined in section IV.D based on Census data. Multi-group horizontal inequality as defined in section V.A.

* $p < 0.1$, ** $p < 0.05$, *** $p < 0.01$. Standard errors in parentheses.

S4 Table. Displacement in communities: Alternative HI measures

	A	B	C	D
	b/se	b/se	b/se	b/se
Uzb-Kyr HI education 0% (0-1)	.2**			
	(.097)			
Uzb-Kyr HI education 5% (0-1)		.65***		
		(.23)		
Uzb-Kyr HI education 10% (0-1)			.65***	
			(.24)	
M-group HI education for Uzb-Kyr (0-1)				.91**
				(.45)
Gini years of education (0-1)	.24	.34	.48	.17
	(.32)	(.39)	(.38)	(.33)
Gini education Uzbeks (0-1)	.15	.19	.14	.22
	(.32)	(.29)	(.29)	(.31)
Gini education Kyrgyz (0-1)	-.044	-.14	-.21	.0062
	(.24)	(.32)	(.31)	(.26)
Ethnic polarization (0-1)	-.0056	-.044	-.04	-.052
	(.028)	(.033)	(.033)	(.042)
Controls	Yes	Yes	Yes	Yes
R2	.23	.38	.38	.29
Observations	120	120	120	120

Note: Sample based on 120 sampling communities in LiK study. Coefficients are from OLS regressions. Standard errors are robust. The dependent variable is the fraction of displaced population from the total population. Horizontal inequality defined in equation (1) with Census 2009 data. Vertical inequality are gini coefficients as defined in section IV.D based on years of completed schooling from the LiK 2010 study. Ethnic polarization as defined in section IV.D based on Census data. Multi-group horizontal inequality as defined in section V.A.

* p < 0.1, ** p < 0.05, *** p < 0.01. Standard errors in parentheses.

S5 Table. Displacement in communities: Always lived in current community sample

	A	B	C	D	E	F
	b/se	b/se	b/se	b/se	b/se	b/se
Uzb.-Kyr. HI education 2% (0-1)	.52***	.52***	.53***	.54***	.59***	
	(.18)	(.18)	(.18)	(.18)	(.21)	
Multi-group HI education (0-1)						.44
						(.46)
Gini years of education (0-1)		.2		.23	.26	.15
		(.21)		(.37)	(.38)	(.34)
Gini education Uzbeks (0-1)			.35	.34	.31	.36
			(.28)	(.27)	(.27)	(.31)
Gini education Kyrgyz (0-1)			.059	-.09	-.12	-.019
			(.14)	(.3)	(.3)	(.27)
Ethnic polarization (0-1)					-.038	-.015
					(.03)	(.036)
Controls	Yes	Yes	Yes	Yes	Yes	Yes
R2	.34	.35	.36	.37	.38	.25
Observations	120	120	120	120	120	120

Note: Sample based on 120 sampling communities in LiK study. The outcome measures the fraction of displaced among individuals who have always lived in the current community. Coefficients are from OLS regressions. Standard errors are robust. Horizontal inequality defined in equation (1) with Census 2009 data. Vertical inequality are gini coefficients as defined in section IV.D based on years of completed schooling from the LiK 2010 study. Ethnic polarization as defined in section IV.D based on Census data. Multi-group horizontal inequality as defined in section V.A.

* p < 0.1, ** p < 0.05, *** p < 0.01. Standard errors in parentheses.

S6 Table. Forced displacement in communities: Weighted by Uzbek population share

	A	B	C	D	E	F
	b/se	b/se	b/se	b/se	b/se	b/se
Uzb-Kyr HI education (0-1)	.82**	.82**	.71*	.74*	.52	
	(.32)	(.32)	(.4)	(.41)	(.44)	
Multi-group HI education (0-1)						.55
						(.84)
Gini years of education (0-1)		2.3		5**	5.6**	5.6**
		(1.5)		(2.2)	(2.3)	(2.4)
Gini education Uzbeks (0-1)			-1.2	-2.2*	-2.4**	-2.4*
			(1.3)	(1.2)	(1.2)	(1.2)
Gini education Kyrgyz (0-1)			2.6*	.077	-.43	-.41
			(1.4)	(.87)	(1)	(1)
Ethnic polarization (0-1)					-.34**	-.4***
					(.16)	(.15)
Controls	Yes	Yes	Yes	Yes	Yes	Yes
R2	.39	.47	.5	.62	.64	.63
Observations	106	106	106	106	106	106

Note: Sample based on 120 sampling communities in LiK study. Coefficients are average marginal effects reported from OLS regressions. Standard errors are robust. The dependent is the fraction of displaced population from the total population. Horizontal inequality defined in equation (1) with Census 2009 data. Vertical inequality are gini coefficients as defined in section IV.D based on years of completed schooling from the LiK 2010 study. Ethnic polarization as defined in section IV.D based on Census data. Multi-group horizontal inequality as defined in section V.A.

* $p < 0.1$, ** $p < 0.05$, *** $p < 0.01$. Standard errors in parentheses.

S7 Table. Forced displacement in communities: Weighted by Kyrgyz population share

	A	B	C	D	E	F
	b/se	b/se	b/se	b/se	b/se	b/se
Uzb-Kyr HI education (0-1)	.71***	.68***	.74***	.71***	.8***	
	(.25)	(.24)	(.25)	(.25)	(.3)	
Multi-group HI education (0-1)						.89*
						(.52)
Gini years of education (0-1)		1.2**		.95	1	.95
		(.48)		(.76)	(.77)	(.81)
Gini education Uzbeks (0-1)			-.17	-.22	-.24	-.2
			(.29)	(.27)	(.27)	(.28)
Gini education Kyrgyz (0-1)			.91***	.39	.33	.34
			(.33)	(.44)	(.46)	(.47)
Ethnic polarization (0-1)					-.085	-.048
					(.077)	(.081)
Controls	Yes	Yes	Yes	Yes	Yes	Yes
R2	.52	.56	.56	.57	.58	.55
Observations	120	120	120	120	120	120

Note: Sample based on 120 sampling communities in LiK study. Coefficients are average marginal effects reported from OLS regressions. Standard errors are robust. The dependent is the fraction of displaced population from the total population. Horizontal inequality defined in equation (1) with Census 2009 data. Vertical inequality are gini coefficients as defined in section IV.D based on years of completed schooling from the LiK 2010 study. Ethnic polarization as defined in section IV.D based on Census data. Multi-group horizontal inequality as defined in section V.A.

* $p < 0.1$, ** $p < 0.05$, *** $p < 0.01$. Standard errors in parentheses.

S8 Table. Forced displacement in communities: Weighted by total population share

	A b/se	B b/se	C b/se	D b/se	E b/se	F b/se
Uzb-Kyr HI education (0-1)	.72***	.68***	.75***	.71***	.77***	
	(.24)	(.23)	(.24)	(.24)	(.26)	
Multi-group HI education (0-1)						.88*
						(.51)
Gini years of education (0-1)		1.5***		1.4*	1.4*	1.3*
		(.49)		(.76)	(.79)	(.8)
Gini education Uzbeks (0-1)			-.19	-.28	-.29	-.26
			(.32)	(.31)	(.31)	(.32)
Gini education Kyrgyz (0-1)			1***	.29	.23	.27
			(.32)	(.4)	(.43)	(.44)
Ethnic polarization (0-1)					-.072	-.056
					(.073)	(.076)
Controls	Yes	Yes	Yes	Yes	Yes	Yes
R2	.52	.57	.56	.58	.59	.56
Observations	120	120	120	120	120	120

Note: Sample based on 120 sampling communities in LiK study. Coefficients are average marginal effects reported from OLS regressions. Standard errors are robust. The dependent is the fraction of displaced population from the total population. Horizontal inequality defined in equation (1) with Census 2009 data. Vertical inequality are gini coefficients as defined in section IV.D based on years of completed schooling from the LiK 2010 study. Ethnic polarization as defined in section IV.D based on Census data. Multi-group horizontal inequality as defined in section V.A.

* p < 0.1, ** p < 0.05, *** p < 0.01. Standard errors in parentheses.

S9 Table. Individual displacement: Always lived in current community sample

	A b/se	B b/se	C b/se	D b/se	E b/se
Education(completed years)	.00042	-.00013	-.0011	-.00025	-.0002
	(.00078)	(.00098)	(.00091)	(.001)	(.00092)
Uzbek (No=0,Yes=1)	.057***	.066***	.11***	.12***	.16***
	(.012)	(.021)	(.032)	(.038)	(.046)
Krygyz (No=0,Yes=1)	.0058	-.0036	-.0095	-.0027	.011
	(.0051)	(.0088)	(.0081)	(.0098)	(.013)
1(edu > p^{uz})		-.012	-.032	-.029	.015
		(.024)	(.021)	(.022)	(.028)
1(edu > p^{ky})		-.0099*	-.011**	.0083	-.00048
		(.006)	(.0048)	(.0078)	(.0081)
HI: Uzbek * 1(edu > p^{ky})		-.0012	-.039	-.064	-.12**
		(.029)	(.038)	(.043)	(.053)
HI: Kyrgyz * 1(edu > p^{uz})		.031	.047**	.018	-.012
		(.021)	(.019)	(.019)	(.024)
Controls	Yes	Yes	Yes	Yes	Yes
Threshold statistic	-	20th	40th	60th	80th
Threshold geography j	-	District	District	District	District
Regional control	District	District	District	District	District
N	4958	4958	4958	4958	4958

Note: Linear probability models with binary outcome equal to 1 if individual was displaced. The sample is based on all individual respondents of the LiK study older than 17 years and who have always lived in the community they were surveyed in. Positional inequality dummy 1(edu > t) are as defined in equation 2. As thresholds we use quintile values as specified in row Threshold stat. Controls are distance to Uzbek border, age bins, gender, nr of children, married, parental education background, self-employment, born in community and landowner. All specifications include dummy variables for each district.

* p < 0.1, ** p < 0.05, *** p < 0.01. Standard errors in parentheses.

S10 Table. Forced displacement by type

	A	B	C	D
	IDPs	IDPs	Refugees	Refugees
	b/se	b/se	b/se	b/se
Education(completed years)	.00022	-.0011	-.000063	.00045
	(.00059)	(.00094)	(.00017)	(.00028)
Uzbek (No=0,Yes=1)	.017*	.045*	.018***	.012
	(.0099)	(.024)	(.004)	(.012)
Krygyz (No=0,Yes=1)	.0038	.0065	.0019	.0023*
	(.0034)	(.0042)	(.0013)	(.0012)
1(edu > μ_{uz})		.015		.0057
		(.015)		(.0082)
1(edu > μ_{ky})		-.0099**		.003**
		(.0045)		(.0012)
HI: Uzbek * 1(edu > μ_{ky})		-.036		.0056
		(.027)		(.014)
HI: Kyrgyz * 1(edu > μ_{uz})		-.022		-.0045
		(.015)		(.0074)
Controls	Yes	Yes	Yes	Yes
Threshold statistic	Mean	Mean	Mean	Mean
Threshold geography j	District	District	District	District
Regional control	District	District	District	District
N	7018	7018	7018	7018

Note: Linear probability models with binary outcome equal to 1 if individual was internally displaced (IDP) or fled across the border (refugee). The sample is based on all individual respondents of the LiK study older than 17 years. Positional inequality dummy 1(edu > t) are as defined in equation 2. As thresholds we use district-level mean education $t_j^e = \sum_i^{N_e} \frac{edu_{i,j}}{N_e}$. All specifications include dummy variables for districts.

* p < 0.1, ** p < 0.05, *** p < 0.01. Standard errors in parentheses.
