# Supplementary material for: Ethnic inequality and forced displacement
Source: PLoS One. 2022 Apr 8;17(4):e0266448. doi: 10.1371/journal.pone.0266448 (PMC8992982; doi:10.1371/journal.pone.0266448)
Supplement: Attachment — Submitted filename: Ethnic inequality and conflict.pdf [file pone.0266448.s002.pdf]

Reviewer comments **July 2021**

Thank you for the opportunity to review your interesting paper "Ethnic inequality and forced displacement." The paper uses the 2010 conflict episode in Kurdistan as a case study to explore the role of inequality in the displacement of minority groups during times of conflict. At the heart of the paper is the question of whether displacement becomes more likely when (1) there is high within-group, or *vertical* inequality, or (2) there is high between-group, or *horizontal* inequality. Kyrgyzstan provides a unique case study to explore these questions. Moreover, Central Asia is often overlooked in the forced displacement literature, so this paper helps to fill this gap in geographic scope. The paper carefully discusses data used and limitations, and it operationalizes inequality using well-established indicators used elsewhere in the literature.

Major comments

1. Currently, I think the paper is greatly missing a clear discussion of the channels through which inequality, conflict, *and* displacement are linked and would benefit from a clearer analytical plan for studying these channels. A question inherent to the paper's focus is as follows: is displacement an *indirect* outcome of (vertical or horizontal) inequality-induced conflict, with the displacement episode being proportional to the degree of conflict exposure? Or in times of conflict, does (vertical or horizontal) inequality make displacement more likely?

   In other words, if we have two communities equally exposed to conflict but with different levels of inequality, is the magnitude of displacement similar for both communities, or is it more intensive for the community with higher inequality? And what form of inequality (vertical or horizontal) matters most?

   By clearly describing the nexus of inequality, conflict, and displacement explored in this paper, the paper could better demonstrate its contribution to the literature and its rationale for using displacement as the primary outcome variable.

   If displacement serves as a sort of proxy for conflict in the analysis, then it's unclear why the paper uses the displacement data from the LiK study instead of using the Uppsala data for the outcome variable. Displacement becomes a more interesting outcome if we believe that the same factors that may make conflict more likely (inequality) also make displacement during the conflict even more intense.

   Clarifying these potential inter-linkages will also help augment the analysis. In Table 3, Columns A-E estimate a regression with displacement on the left hand side and inequality measures (and covariates) on the right hand side. The coefficient estimates on the horizontal inequality variable are significant and positive. But isn't it possible that this significant result is entirely driven by conflict exposure, an omitted variable? If

conflict exposure is positively correlated with horizontal inequality (Table 4 Column E) and is also correlated with the share of the population displaced, then the significant result may not really tell us anything meaningful about inequality and displacement, but may instead reflect the relationship between inequality and conflict.

2. Building on (1), I recommend estimating the regression reported in Table 3 (with displacement as an outcome variable) including conflict as a control. I understand that one reason this may not have been part of the original strategy is because conflict and the inequality metrics are correlated. Nevertheless, I feel that this additional regression will help us get a little closer to the question of whether inequality makes displacement more likely, even when we hold the intensity of conflict exposure constant.

3. The paper discusses several potential sources of bias. But I think there is one other item to keep in mind related to mismeasurement of the displacement outcome variable. The paper discusses how some of the displaced may not have returned to their communities. The outcome variable is the share of displaced returnees, and subsequently the share of displaced non-returnees (theoretically) is part of the error term. Is this variable correlated with the inequality variables? If yes, then this is a source of omitted variable bias.

   The paper's discussion of empirical limitations would be stronger if it also discussed measurement error in the outcome variable (for the community level regression) at greater length and incorporated this into the interpretation of the results.

4. According to Table 2, the horizontal inequality (HI) measure between Uzbeks and Kyrgyz education has a rather skewed distribution, with very low HI (less than 0.09) in most provinces, with the exception of Osh City. My concern is that the Osh City respondents may be completely driving the outcome, given the lack of variation. The paper could address this concern by first plotting the distribution of HI scores for all communities. If there are indeed some outliers with HI measures well above 0.1, then the paper would benefit from a robustness test that drops these outliers and examines whether very small differences in HI in areas with rather small HI still matter.

5. For the regression covered in Equation 2, I was surprised by the paper's switch from carefully non-causal language to causal language. Ex: "We interpret the delta coefficients as average effects from between-ethnicity differences in education on the probability to flee." But there is no clear description of an identification strategy that would provide support of a causal interpretation. Equation 2 includes more controls, which reduces the propensity of omitted variables to bias the coefficient estimates. But even with additional covariates, the regression's interpretation is not causal if the regression cannot exploit exogenous variation in the explanatory variable or valid counterfactual estimation. If the

paper can make a strong case for the identification in Equation (2) to be sufficient for causal interpretation, this needs to be made more clearly to the audience. Otherwise I feel the paper should again emphasize that these are important and meaningful correlations that are being examined.

6. Equation 2 introduces some rather complex variables, and I understand that such indicators are difficult to describe in words. But I must admit that I greatly struggled to understand the estimation strategy and interpretation of results for the regressions defined by Equation 2. The first item I had to spend time figuring out is that in $(edu_{ij} > t_j^e)$, the parameter e need not be equal to the respondent's own group. It would really help the reader if this point was made very explicitly and very early.

   I also struggled to bridge Table 5 and the discussion of the results on p. 14-15. The variables defined operate on the basis of being *above* a group's education cutoff for a given quintile. My initial interpretation from the table is that the better off an individual is relative to the other group, the *less* likely they were to experience displacement. Ex: Uzbeks who fall in the 60th percentile of Kyrgyz education or above are 7.2 percentage points less likely to have experienced displacement (Column D). But the paper reports results for groups that fall in the bottom of the other group's education distribution. Ex: "Educated Uzbeks ranked in the bottom 80% of Kyrgyz have a 14 pp higher probability of displacement than other ethnicities." Where does this result come from? What variable provides us with the mean outcome for Uzbeks below (not above) the Kyrgyz quintile cutoff points?

   Finally, I am struggling to understand how the estimations from Equation 2 provide us with more insight about who is displaced when inequality leads to displacement. One could easily interpret these results as providing information on whether wealthier people (as proxied by education) stay or flee at times of conflict. After all, an Uzbek respondent who falls in a higher Kyrgyz education quintile is not just relatively more educated but is also more educated in the absolute sense. The paper needs to clearly justify how these results add to our knowledge of the ethnic inequality-displacement nexus and are not just reflections of whether richer or poorer people experience displacement during conflict episodes.

   I fully acknowledge that some of these questions may be related to my struggle to fully understand the estimation approach. But I feel that this confusion still highlights the need for the paper to more clearly present the regression strategy in Equation 2 more clearly to the audience.

7. Despite my struggles to understand Equation 2, I am again concerned about conflict as an omitted variable. Is conflict exposure included in the vector of covariates? If not, is it possible to re-estimate the results while including conflict exposure? In this case, I worry that the better-off in either group (as proxied by falling in a high educational quintile of the in-group or the out-group) may be less conflict-exposed, making their displacement less likely.

8. In the Introduction, the paper evaluates the result from Regression 2 through the lens of Basu (2005). But I do not think that this is the most appropriate paper to draw on in this case. Basu (2005) focuses on expected prejudice of one out-group member, which is a function of the distribution of prejudice among the entire out-group. I don't think this paper really comments on education as a mechanism. Reconciliation policies enacted through educational institutions may reduce out-group prejudice, but I don't think it will alter how an individual derives the expected prejudice of the out-group.

   I believe that paper would be greatly enhanced by further discussion of why the more educated were less likely to experience displacement. Could this be due to unequal access to institutions such as protective security? Do the higher educated tend to live in areas that were further from the violence? Etc.

Minor comments

1. The Figure 1 map would greatly benefit from some editing. First, I recommend dropping the grid markers for latitude and longitude and dropping the xy axis labels. The reader has enough information from the country lines and the neighboring country locations to understand where they are looking on the globe, and this additional content just makes the map more cluttered.

   Second, I see three variables conveyed: the geographic location of respondent clusters, circles that indicate the magnitude of displacement at a cluster, and a greyscale-coded scale of "HI" (horizontal inequality). I think the map is difficult to read because so much is going on and layers overlap. I recommend producing two maps: one demonstrating the cluster locations - this can include the HI scale - and the other can illustrate the magnitude of displacement by making the cluster location's dot size a function of the magnitude of displacement.

2. I additionally recommend mapping the Uppsala data on Kyrgyzstan for the period as points. This will help the reader see spatial patterns of inequality, conflict, and

displacement. It would also be helpful to have a sense of the number of total conflict events. Combined with the previous comment, this would mean including three maps in the paper: (1) cluster locations and HI scores, (2) magnitudes of displacement, and (3) conflict event locations.

3. Table 2: please transform the rows reporting percentages (row 2 and 3) by multiplying by 100. At present they are proportions, not percentages.

4. For the first econometric specification included in Section V.A (p10), it would help the reader a lot if you could include the econometric specification along with the discussion of the specification. I ended up writing out a version of this in my notes to help me interpret the table.

5. For the first econometric specification (p10), I understand the choice of a GMM estimator, but why include the logit link? Is it possible to estimate based on LPM so that the coefficients are easier to interpret? Please further justify the choice of a logit link or consider estimating with LPM.

6. P10 includes a transformation of one of the coefficient results. In a footnote, please explain to the reader how to perform this transformation. For example, 0.39 is the coefficient estimate, but where does 0.084 come from?

7. In the discussion of Equation 2, please list the variables that make up the vector of covariates. For example, is community distance to the border with Uzbekistan still included?

8. I flagged several typos in the manuscript: I am listing all of those that I noted here, but I also recommend a careful copy-editing of the paper to catch any additional mistakes:
   a. P1 “A short period of violence, left more…” → “A short period of violence left more…”
   b. P2 “The conflict raises the question how racism and ethnic hatred can emerge?”, which could be changed to, “The conflict raises several questions, including: how can racism and ethnic hatred emerge?”
   c. P2 “close to the Uzbek border” → “close to the border with Uzbekistan”
   d. P5 “nowadays Kyrgyzstan” → “contemporary Kyrgyzstan”
   e. P5 “...the context as follows: The global..” → “...the context as follows: the global…”
   f. P6 “Using the nationally representative LiK study, ...no over-sampling of the temporarily displaced population took place unfortunately.” Please remove “unfortunately”.

g. P10 “does not seem spatially coincide” → “does not seem to spatially coincide”
h. P12 “Column E of table 4 uses replaces displacement…” → “Column E of Table 4 replaces displacement”
i. P14 “...the decision to flee on the individual position in the…” → “...the decision to flee on the individual’s position in the…”
j. P14 “kyrgyz or uzbek” → “Kyrgyz or Uzbek”
k. P15 Table 5 “Treshold” → “Threshold”
l. P15 “Column B and E” I think maybe was meant to be “Columns B through E”

Thank you again for your efforts to explore the nexus of ethnic inequality, conflict, and displacement and to use the unique LiK study to help us build an understanding of the microeconomics of conflict.
