# Supplementary material for: Ethnic inequality and forced displacement
Source: PLoS One. 2022 Apr 8;17(4):e0266448. doi: 10.1371/journal.pone.0266448 (PMC8992982; doi:10.1371/journal.pone.0266448)
Supplement: Attachment — Submitted filename: reply_to_referees.pdf [file pone.0266448.s003.pdf]

Response to the reviewers

We would like to thank the reviewers for the thorough review of our work. In the following, we would like to address their concerns point by point. We completely revised the manuscript on the basis of their comments. We believe that the thoughtful criticism helped to improve the manuscript considerably.

Please kindly take note that we uploaded all codes and datasets (except for the LiK Study which is open access but requires personal registration) to this anonymous webarchive [link] accessible with the password “displacement2010”.

Reviewer 1

Reviewer Point P 1.1 — Currently, I think the paper is greatly missing a clear discussion of the channels through which inequality, conflict, and displacement are linked and would benefit from a clearer analytical plan for studying these channels. A question inherent to the paper's focus is as follows: is displacement an indirect outcome of (vertical or horizontal) inequality-induced conflict, with the displacement episode being proportional to the degree of conflict exposure? Or in times of conflict, does (vertical or horizontal) inequality make displacement more likely?

In other words, if we have two communities equally exposed to conflict but with different levels of inequality, is the magnitude of displacement similar for both communities, or is it more intensive for the community with higher inequality? And what form of inequality (vertical or horizontal) matters most?

By clearly describing the nexus of inequality, conflict, and displacement explored in this paper, the paper could better demonstrate its contribution to the literature and its rationale for using displacement as the primary outcome variable. If displacement serves as a sort of proxy for conflict in the analysis, then it's unclear why the paper uses the displacement data from the LiK study instead of using the Uppsala data for the outcome variable. Displacement becomes a more interesting outcome if we believe that the same factors that may make conflict more likely (inequality) also make displacement during the conflict even more intense.

Clarifying these potential inter-linkages will also help augment the analysis. In Table 3, Columns A-E estimate a regression with displacement on the left hand side and inequality measures (and covariates) on the right hand side. The coefficient estimates on the horizontal inequality variable are significant and positive. But isn't it possible that this significant result is entirely driven by conflict exposure, an omitted variable? If conflict exposure is positively correlated with horizontal inequality (Table 4 Column E) and is also correlated with the share of the population displaced, then the significant result may not really tell us anything meaningful about inequality and displacement, but may instead reflect the relationship between inequality and conflict.

Reply: We are grateful about this comment. Rereading our paper, we absolutely agree with Referee 1 on this point and Referee 2's comment P 2.3 that the paper's theoretical assumptions needed to be made explicit and developed. We tried to take both of their comments to our hearts and therefore took the decision to replace the literature review with a theoretical framework grounded in our reading of the existing literature. We believe that violence and forced displacement are both an outcome of inequalities. Even though we currently lack causal evidence on these relationships, the existing literature with a few exceptions agrees theoretically on this that disparities in income/wealth/education across and within

different ethnicities can cause forced displacement and violence. Figure 1 encodes these causal relationships underpinning our thinking based on our reading of the current state of the literature. Regressing inequalities on displacement without violence estimates therefore the total association. Violence would not be an omitted variable as we conjecture that it does not impact pre-conflict inequalities, it's the other way around. Including violence into the regression would make it a "bad control" as long as we are interested in the total association (Model 11 in Cinelli et al. [2020]). If we were interested exactly in the controlled direct association of inequalities on displacement holding conflict constant, then we should control for it. However, this might come at the cost of confounders impacting violence and displacement, creating even more endogeneity than needed (see Cinelli and coauthors for the graph).

Reviewer Point P 1.2 — Building on (1), I recommend estimating the regression reported in Table 3 (with displacement as an outcome variable) including conflict as a control. I understand that one reason this may not have been part of the original strategy is because conflict and the inequality metrics are correlated. Nevertheless, I feel that this additional regression will help us get a little closer to the question of whether inequality makes displacement more likely, even when we hold the intensity of conflict exposure constant.

Reply: We included a discussion based on your thoughts in the results section and report results of the mediated association with violence in Table 4:

> *We estimate the direct and indirect association through distance to violence of forced displacement with inequalities in Table 4. The correlation between HIs and displacement decreases by 6pp, which could be interpreted under the strong assumption, that no other confounders correlated with violence and displacement introduce bias that most of the total association between HIs and displacement is indeed direct.*

Reviewer Point P 1.3 — The paper discusses several potential sources of bias. But I think there is one other item to keep in mind related to mismeasurement of the displacement outcome variable. The paper discusses how some of the displaced may not have returned to their communities. The outcome variable is the share of displaced returnees, and subsequently the share of displaced non-returnees (theoretically) is part of the error term. Is this variable correlated with the inequality variables? If yes, then this is a source of omitted variable bias. The paper's discussion of empirical limitations would be stronger if it also discussed measurement error in the outcome variable (for the community level regression) at greater length and incorporated this into the interpretation of the results.

Reply: We had to think hard about this comment. This is what we believe is going on with measurement error and what we can do against it in the section of the community results:

> *Another concern ist that our displacement outcome might suffer from measurement error. Random measurement error would attenuate the coefficient down to zero. If displaced individuals did not return to their place of residence during the conflict because of ethnic inequalities, measurement error would act like an omitted variable bias. Table 10 reports results with the fraction of displaced among those who indicate to have never lived somewhere else. This subsample of the population is certainly different in many aspects, but estimated coefficients are very similar in magnitude and significance. This could be interpreted that*

bias from returning to live somewhere else in Kyrgyzstan after forced displacement must be limited. Moreover, there might be more non-returnees to Kyrgyzstan in conflict-prone communities. This could create confounding correlated with ethnic inequalities as well. To quantify the sensitivity of these results with respect to omitted variables we report the battery of statistics suggested by Cinelli and Hazlett [2020] for the regression in Table 3.

Reviewer Point P 1.4 — According to Table 2, the horizontal inequality (HI) measure between Uzbeks and Kyrgyz education has a rather skewed distribution, with very low HI (less than 0.09) in most provinces, with the exception of Osh City. My concern is that the Osh City respondents may be completely driving the outcome, given the lack of variation. The paper could address this concern by first plotting the distribution of HI scores for all communities. If there are indeed some outliers with HI measures well above 0.1, then the paper would benefit from a robustness test that drops these outliers and examines whether very small differences in HI in areas with rather small HI still matter.

Reply: We share this concern. We therefore added a scatterplot in Figure 5 and regression Table 8 excludes Osh and Osh city as a robustness check. The results are reassuring.

Reviewer Point P 1.5 — For the regression covered in Equation 2, I was surprised by the paper's switch from carefully non-causal language to causal language. Ex: "We interpret the delta coefficients as average effects from between-ethnicity differences in education on the probability to flee." But there is no clear description of an identification strategy that would provide support of a causal interpretation. Equation 2 includes more controls, which reduces the propensity of omitted variables to bias the coefficient estimates. But even with additional covariates, the regression's interpretation is not causal if the regression cannot exploit exogenous variation in the explanatory variable or valid counterfactual estimation. If the paper can make a strong case for the identification in Equation (2) to be sufficient for causal interpretation, this needs to be made more clearly to the audience. Otherwise I feel the paper should again emphasize that these are important and meaningful correlations that are being examined.

Reply: The referee is absolutely right in pointing out the misplaced use of language here. We agree that the use of the term "effect" invokes a causal interpretation when used contemporarily, even though the word effect does not necessarily imply a causal effect historically in econometrics and statistics (e.g. average marginal effects/AMEs in probit models). We now instead use association and correlation throughout the paper.

Reviewer Point P 1.6 — Equation 2 introduces some rather complex variables, and I understand that such indicators are difficult to describe in words. But I must admit that I greatly struggled to understand the estimation strategy and interpretation of results for the regressions defined by Equation 2. The first item I had to spend time figuring out is that in $(edu_{ij} > t_j)$ parameter e need not be equal to the respondent's own group. It would really help the reader if this point was made very explicitly and very early.

Reply: This would be possible to do, to include a dummy for an individual being highly educated compared to her own ethnicity. But as horizontal inequalities seem to be the associated driver in the community regressions, we decided to decomplexify and focus only on disparities between both ethnic groups. We elaborated this point as follows:

The dummy variable $\mathbb{1}(\text{edu}_{\text{i,j}} > t_j^e)$ in equation 3 indicates whether individual i with education level edu_i is above a threshold t_j^e on the distribution of education of the other ethnic group e (Kyrgyz or Uzbek) in area j. What we want to estimate is whether e.g. Uzbeks who are either more or less educated than most Kyrgyz in their district might have different probabilities of fleeing controlling for the general influence of education.

I also struggled to bridge Table 5 and the discussion of the results on p. 14-15. The variables defined operate on the basis of being above a group's education cutoff for a given quintile. My initial interpretation from the table is that the better off an individual is relative to the other group, the less likely they were to experience displacement. Ex: Uzbeks who fall in the 60th percentile of Kyrgyz education or above are 7.2 percentage points less likely to have experienced displacement (Column D). But the paper reports results for groups that fall in the bottom of the other group's education distribution. Ex: "Educated Uzbeks ranked in the bottom 80% of Kyrgyz have a 14 pp higher probability of displacement than other ethnicities." Where does this result come from? What variable provides us with the mean outcome for Uzbeks below (not above) the Kyrgyz quintile cutoff points?

Reply: We agree that this is not a great exposition of the results. The dummy indicates that an Uzbek would rank above the bottom 80% of Kyrgyz. This could be read the other way around that an Uzbek below the top 20% of Kyrgyz does not fall in this category. The total correlation of being Uzbek in the bottom 80% of Kyrgyz would only be $\hat{\beta_1} = 14pp$ compared to other ethnicities without adding the coefficient δ_1. We now explain coefficients as follows:

Introducing horizontal inequality, columns B to E reveal how horizontal disadvantage in education towards the other ethnic groups manifests itself in an associated increase in the likelihood to flee. On the other hand, horizontal advantage of Uzbeks and Krygyz vis à vis the other ethnic group is associated with a lower probabibilty to flee. Uzbeks better educated than the bottom 80% of Kyrgyz in the district are $\delta_1 = -12pp$ less likely to be displaced than Uzbeks with education levels comparable to the bottom 80% of Kyrgyz. The same direction of correlation holds true for ethnic Kyrgyz who are $\delta_2 = -2.5pp$ less likely to be displaced above than below the 80th percentile of Uzbek education in their district.

Further:

Conversely, Uzbeks ranked in the bottom 80% of Kyrgyz have a $\beta_1 + \delta_1 = 14pp$ higher probability of displacement than other ethnicities. Educated Kyrgyz ranked in the bottom 80% of Uzbeks have a $\beta_2 + \delta_2 = 1.8pp$ higher probability of displacement than other ethnicities. This means that highly ranked Uzbeks and Kzrgyz vis a vis the other ethnic group were significantly more likely compared to their lower ranked counterparts. The association of educational advantage over Kyrgyz oder Uzbeks does not hold for other ethnic groups as evidenced by the insignificant coefficients γ_1 and γ_2.

Finally, I am struggling to understand how the estimations from Equation 2 provide us with more insight about who is displaced when inequality leads to displacement. One could easily interpret these results as providing information on whether wealthier people (as proxied by education) stay or flee at times of conflict. After all, an Uzbek respondent who falls in a higher Kyrgyz education quintile is not just relatively more educated but is also more educated in the absolute sense. The

paper needs to clearly justify how these results add to our knowledge of the ethnic inequality-displacement nexus and are not just reflections of whether richer or poorer people experience displacement during conflict episodes.

Reply: We believe that these individual regressions are insightful because they can reveal who actually fled as opposed to the community regressions. The fact that not education per se, but being higher educated than people of the other ethnicity in my district is associated with displacement adds a novel nuance in our understanding of forced displacement.

Reviewer Point P 1.7 — I fully acknowledge that some of these questions may be related to my struggle to fully understand the estimation approach. But I feel that this confusion still highlights the need for the paper to more clearly present the regression strategy in Equation 2 more clearly to the audience.

Reply: Based on your thorough comments we rewrote this section for the new version. We hope that these changes improved overall readability.

Reviewer Point P 1.8 — Despite my struggles to understand Equation 2, I am again concerned about conflict as an omitted variable. Is conflict exposure included in the vector of covariates? If not, is it possible to re-estimate the results while including conflict exposure? In this case, I worry that the better-off in either group (as proxied by falling in a high educational quintile of the in-group or the out-group) may be less conflict-exposed, making their displacement less likely.

Reply: Refering to the discussion under point P 1.1 again, we argue that conflict is not an omitted variable under the assumptions in the DAG rather a mediator (or "channel"). We present regressions controlling for violence in Table 17. However, we cannot control for individual distance within a community. The coefficient is estimated on community-level variation within districts. Against this background, one might conclude again that the direct association between inequalities and forced displacement seems not to be affected much by controlling for the mediator violent conflict.

Reviewer Point P 1.9 — In the Introduction, the paper evaluates the result from Regression 2 through the lens of Basu (2005). But I do not think that this is the most appropriate paper to draw on in this case. Basu (2005) focuses on expected prejudice of one out-group member, which is a function of the distribution of prejudice among the entire out-group. I don't think this paper really comments on education as a mechanism. Reconciliation policies enacted through educational institutions may reduce out-group prejudice, but I don't think it will alter how an individual derives the expected prejudice of the out-group. I believe that paper would be greatly enhanced by further discussion of why the more educated were less likely to experience displacement. Could this be due to unequal access to institutions such as protective security? Do the higher educated tend to live in areas that were further from the violence? Etc.

Reply: We offer the following revised explanation for our findings from the individual regressions:

> *The scapegoat theory as advanced by Bramoullé and Morault [2016] does not square off with our results as we find that Uzbeks (and Kyrgyz) are less likely to leave when they constitute the local educational elites. We reconcile our findings with the narrative that in places where two ethnicities compete over access to public resources (education), the people*

who flee are most likely to have fewer opportunities. Moreover, the educated elite on both sides could be inclined to stay in spite of the risks, as they would fear to lose their privileges to the other ethnic group. This view would be supported by the fact that controlling for violence does not change the estimated coefficients. The risks associated with flight might have outweighed the risks from staying for the educated elites.

Minor

Reviewer Point P 1.10 — Maps: grid markers

Reply: Taken out from graphs

Reviewer Point P 1.11 — Maps: ...(1) cluster locations and HI scores, (2) magnitudes of displacement, and (3) conflict event locations.

Reply: See Figure 2

Reviewer Point P 1.12 — Table 2: Percentages instead of proportions

Reply: Corrected

Reviewer Point P 1.13 — include the econometric specification along with the discussion of the specification

Reply: Included

Reviewer Point P 1.14 — estimate based on LPM

Reply: Corrected

Reviewer Point P 1.15 — transformation of one of the coefficient results

Reply: Not applicable any more

Reviewer Point P 1.16 — In the discussion of Equation 2, please list the variables that make up the vector of covariates. For example, is community distance to the border with Uzbekistan still included?

Reply: Covariates are listed

Reviewer Point P 1.17 — Typos

Reply: Corrected

Reviewer 2

Reviewer Point P 2.1 — The paper has no theory. From the beginning, the authors talk about the importance of ethnicity in conflict but fail to provide a clear theoretical link between inequality in education observed across ethnic lines and forced displacement. This makes the paper extremely confusing and leaves the reader wondering why we should observe any correlation at all. This is further complicated by the fact that the authors list an extensive list of papers about ethnicity and conflict, but do not discuss at all how they relate to their work, or what they contribute to the literature. I suggest that the authors add a theory section where they lay out what is the relationship between the variables they discuss. It seems to me (but I may be confused) that the authors have in mind a causal process that goes from ethnicity to conflict, and has displacement as an outcome, however, the relationship is mediated by education.

Reply: We thank the referee of providing such a clear criticism of the theoretical approach in the previous manuscript. Referee 1 equally pointed out the lack of theoretical grounding. We worked hard to incorporate the points raised by both referees and exchanged the literature section for a theoretical section. Based on our reading of the literature we present a directed acyclic graph as a graphical description of the causal process we have in mind.

Reviewer Point P 2.2 — Laying out these links could also be helpful to note that there is an obvious confounder that affects both inequality in education and the probability of being displaced, which is barely mentioned: income. Several scholars have written extensively about this relationship, and about ethnic minorities and displacement in Colombia, and none are mentioned. Some starting points could be the work of Ana Maria Ibañez or Abbey Steele. More on data later, but the authors should control for income.

Reply: We are thankful about the references to the two authors and their coauthors, whose work greatly informs the new theoretical section. On the use of income as a dimension of inequality we incorporated the following:

> *We measure inequalities in the dimension of education for the pragmatic reason that we do not have pre-conflict information on income or wealth.*

We further motivate the use of education as follows:

> *One concern about this graph might be the directionality of the relationship between conflict and education. For instance, Fergusson et al. [2020]) study the association of conflict with educational attainment. As we are not aware of any research demonstrating a response of education to the expectation of future conflict, we think that completed education pre-conflict might not suffer from simultaneity bias.*

Reviewer Point P 2.3 — Additionally, there are a lot of things we don't know about the context that could be useful to understand how income affects inequality and vice-versa (v.gr. do Uzbeks have schools where they speak their own language? Is it hard to integrate into the labor market if you don't speak the local language?). Moreover, a theory could be helpful to understand the

mechanisms that are underpinning the relationship, because the reader is unable to grasp what is going on: are the less educated being targeted because there are poorly educated and forced to leave? Are the less educated being targeted because they are poor (and being poorly educated is correlated with income)? Are they not being targeted, and leaving out of their own volition to avoid risks? Are they living because they are afraid that they don't have access to the judicial system? Because we don't understand what is going on, the empirical exercise is left to stand alone, and the reader is obliged to make sense of the results. Although in the empirical section the authors affirm, they are not making causal claims, the paper is written as if they were until that section. A theoretical explanation of why income is not a confounder should alleviate that concern partly. I recommend that a proper (and by this, I don't mean necessarily formal) theoretical framework is laid out after the context is presented to explain the relationship of the main variables of interest (is education a moderator??), and the main mechanisms.

Reply: We are thankful about these guiding questions by referee 2 to sharpen the explanation of our findings. We added following discussion of the results of our communitiy cross-section:

> *In summary,* horizontal inequality *emerges as a robust predictor of displacement across communities in the Kyrgyz context. These results do not support Esteban and Ray [2008]'s model, where conflict arises from within-ethnic-group inequality splitting an ethnic group into rich providers of financial means and poor suppliers of conflict labor. We would rather argue along the lines of Murshed and Gates [2005] that on the one hand, the collective experience of deprivation from access to basic and higher education might spur protest mobilization. On the other hand, privileged access to education provided on the basis of ethnicity might strengthen it's importance for identity. Our results are suprising in the sense that Kyrgyzstan has a relatively upward mobile education system in international comparison [Brück and Esenaliev, 2018]. Education is publicly financed and and there exists for example a nationwide policy of free school meals[Muktarbek kyzy, 2019]. Notwithstanding Kyrgyzstan's historically egalitarian education system, one might argue that competition through more accessible education might make discrimation on the basis of ethnicity even more attractive to a local elite to maintain their status. In anecdotal evidence, Canning [2020] describes how Uzbek was scrapped as an official language to take state exams in a process he describes as the "Kyrgyzification" of the teaching system.*

Reviewer Point P 2.4 — It is hard to understand how the data being used can capture what the author is trying to measure. In particular, the data is post-conflict: don't we expect the makeup of the population left behind to be very different from the population pre-conflict? In particular: suppose the wealthy left, aren't we not overestimating the responses from the poor (and less educated) left behind? I believe the authors should discuss how this is not biasing the results, although I can't think of any process that would not generate sample selection bias. If the authors believe the conflict-affected families randomly, and that a random subset of the Uzbeks was left behind such that we can say something that generalizes, they should discuss it.

Reply: This is a great point and referee 1's point P 1.3 seems to agree on this issue being problematic. It is evident that we cannot go back in time to collect pre-conflict data. As the LiK is nationally representative it does capture all forced displaced who returned to Kyrgyzstan, which is an advantage compared to other data sources used throughout the literature. The UNHCR states that most Uzbek

refugees returned shortly after the violence subsided. We unfortunately found no disaggregate data of emigration flows such that we could gauge potential changes in the composition of migration flows out of Kyrgyzstan. We discuss measurement error and sample selection bias as follows:

> *Another concern ist that our displacement outcome might suffer from measurement error. Random measurement error would attenuate the coefficient down to zero. If displaced individuals would not return to their place of residence during the conflict because of ethnic inequalities, measurement error would act like an omitted variable bias. Table 10 reports results with the fraction of displaced among those who indicate to have never lived somewhere else. This subsample of the population is certainly different in many aspects, but estimated coefficients are very similar in magnitude and significance. This could be interpreted that bias from returning to live somewhere else in Kyrgyzstan after forced displacement must be limited. Moreover, there might be more non-returnees to Kyrgyzstan in conflict-prone communities. This could create confounding correlated with ethnic inequalities as well. To quantify the sensitivity of these results with respect to omitted variables we report the battery of statistics suggested by Cinelli and Hazlett [2020] for the regression in Table 3.*

Now, if we suppose the wealthy did not return to their communities at random, this would not create an issue for our estimation. If the wealthy only left the country from communities where violence occured, this would in our understanding act like an omitted variable correlated with violence and inequalities we cannot control for. We test the sensitivity of our estimates in our community regressions, which show that the effect of horizontal inequalities cannot be easily explained away.

For the individual results, suppose wealthy people are more educated and fled the country from conflict areas more often. That might not necssarily pose a problem to the current strategy with which we find that more educated people stayed relative to the other ethnicity's education distribution. If wealthy people fled abroad they would be missing in the distribution of both ethnicities. What we have to assume is that ethnic Kyrgyz and Uzbek would not flee abroad/emigrate differentially.

Reviewer Point P 2.5 — The authors should discuss in what ways the measure HI is not problematic, given that the measure does not account for different group sizes. Maybe they could weigh the districts by group size? Moreover, the authors should explain why then they seem to test inequality among multiple groups with a new measure that does give group size importance. Why not use the same original measure and control by each group's measure?

Reply: We agree with referee 2 that this may create situations where a few individuals can determine the HI index. However, this might actually be desirable in situations where a few individuals were scapegoated or witchhunted for their otherness. To demonstrate robustness of the index against outliers, we included following tables in the results section:

> *Outliers might be a concern suggested by the spatial concentration of violence and displacement in Figure 2. Table 8 in the appendix shows the robustness of associations in our base specification without Osh and Osh city. Moreover, the horizontal inequality index does not account for differences in group size. This can lead to extreme outcomes if for instance only one Uzbek lived in a community and her education would enter the index. We therefore assume an HI of 0 for communities where the Uzbek group size is below 2% in*

our base specfications. Nevertheless, in Table 9 we document that the positive significant correlation is robust to using alternative group thresholds of 0%, 5% and 10% and using the group size-weighted multi-group measure of Table 3 column F for only Kyrygz and Ubzeks. Moreover, Table 11, Table 12 and Table 13 in the Appendix weigh the baseline results with the community share of ethnic Uzbeks and Kyrgyz of total Uzbek and Kyrgyz population and the share of total national population. Our qualitative interpretations remain by and large the same.

Reviewer Point P 2.6 — A special mention goes to the fact that none of the figures or tables are references, and that the famous Figure 1 is either absent or the map presented at the end, which does not show what the authors claim it shows, as there are no labels and it is very hard to make sense of.

Reply: We followed referee 1's advice and reorganized the map. The new manuscript uses clickable references to figures and tables.

Reviewer Point P 2.7 — The explanation for using a glm model is not clear: there are several corrections to estimate standard errors when errors are not normally distributed using OLS. The authors should try to implement these corrections and show an OLS estimation as robustness. The model used in the individual displacement section seems more convincing.

Reply: We now report OLS regression with robust standard errors (HC3 in Stata).

Reviewer Point P 2.8 — The authors should interpret the coefficients in the probability of staying for educated groups of both predominant ethnicities since this brings even more doubts: the fact that both educated Uzbeks and Kyrgyz are more likely to stay makes the reader wonder if what you are showing is not a correlation between income and displacement, which is very likely to be the case.

Reply: One could make the case that ethnic disparities in education actually lead to ethnic disparities in income. Income inequality would then be a mediator between education inequalities and displacement. If we were interested in the total association of educational inequalities we would not control for income inequality. We could control for income inequality to estimate the direct association, but this would in turn invite more omitted variables correlated with income inequality and displacement to bias all coefficients. As post-conflict income is certainly affected by displacement, likely much more than completed education of adults, we strongly think including income would be a bad choice if our overall strategy is to minimize bias.

Reviewer Point P 2.9 — The paper should be edit for clarity.

Reply: We gave our best to improve the paper's readability. We thank referee 2 for the thought-provoking comments and points raised to improve the manuscript.

References

C. Cinelli and C. Hazlett. Making sense of sensitivity: Extending omitted variable bias. *Journal of the Royal Statistical Society: Series B (Statistical Methodology)*, 82(1):39–67, 2020.

L. Fergusson, A. M. Ibáñez, and J. F. Riaño. Conflict, educational attainment, and structural transformation: La violencia in colombia. *Economic Development and Cultural Change*, 69(1): 335–371, 2020. doi: 10.1086/702995. URL `https://doi.org/10.1086/702995`.
