# Supplementary material for: Ethnic inequality and forced displacement
Source: PLoS One. 2022 Apr 8;17(4):e0266448. doi: 10.1371/journal.pone.0266448 (PMC8992982; doi:10.1371/journal.pone.0266448)
Supplement: Attachment — Submitted filename: rebuttal.pdf [file pone.0266448.s004.pdf]

Response to the reviewers

We would like to thank the reviewers for acknowleding the work which went into the revision of the paper based on the first round of comments. Based on a second round of comments by the referees, we would like to lay out in the following our answers and revisions point by point. We believe that the rigorous comments by the referees contributed to a further improvement of the manuscript.

Beyond the comments by the referees, we changed the manuscript with respect to these two issues:

- We excluded the redundant coefficient plot as we present the results in the table already.
- We corrected reported magnitudes in the interpretations of coefficients.

Please kindly take note that all codes and datasets (except for the LiK Study) can be accessed through our anonymous webarchive [link] accessible with the password "displacement2010".

Reviewer 1

Reviewer Point P 1.1 —
The literature review and theory sections have improved tremendously. I have one suggestion for enhancing this section further. One of the determinants of conflict behavior often discussed in economics is the opportunity cost of violence (a concept with origins in Gary Becker's work on crime). I wonder if, given the use of education to operationalize inequality, the opportunity cost of violence may also play a role in understanding the relationship between horizontal inequality and conflict. For example, could the group that has received fewer educational opportunities have a lower opportunity cost of violence? Drawing that literature into the paper's literature review could further enhance the manuscript.

Reply: We would like to thank the referee for acknowledging the hard work which went it these sections. We are grateful about the hint to this influential literature. We added following sentence to the theoretical discussion of mechanism M1:

> *Thirdly, opportunity costs of enganging into violent acts such as looting might be lower for horizontally disadvantaged individuals.*

Reviewer Point P 1.2 —
Overall, the code used to render the manuscript (in Latex, I am guessing?) will need to go through a careful edit in order to ensure that sections are in their appropriate position and tables are not repeated. I was able to review two separate versions, a new clean manuscript and a manuscript showing tracked changes. I focused on the latter, as it helped me understand where changes had taken place. But in both versions, there were some organizational issues in terms of where tables, figures, and sometimes even sections appeared, as well as some repetition of Tables (ex: Table 3 and 4 in the track-edits version are the same?). It's difficult for me to identify specific

fixes since the problems may be slightly different across the two versions I am reading. Please address this.

Reply: We thank the referee for his careful checks on formatting. The regression results of the community cross-section in Table 3 and 4 were indeed repeated in the paper. We carefully screened the tables again and forced the position of tables in the Appendix.

I consider this a non-essential suggestion (please take it or leave it), but as a reader I was curious about what the authors saw as the future direction of this work on inequality and displacement. What additional questions or ideas did your analysis of Kyrgyzstan spark? Please don't feel obligated to fabricate this part (sometimes the "next steps" section of a paper feels like total fluff), but if the authors have some ideas for future directions to add to the Conclusion, I would be super interested in reading this.

Reply: We added the following sentences to the conclusion:

> *We hope that future work can circumvent some of the identification assumptions in this paper imposed by data availability. Following individuals through the conflict and potential displacement over time with longitudinal data would open a new set of possibilities to study the decision to flee from conflict.*

Minor

Reviewer Point P 1.3 — P. 3 "Northern capital Bishkek" → "northern capital Bishkek"

Reply: Corrected

Reviewer Point P 1.4 — Fig 1 (clean version) formatting issue: with a black background, black text that exceeds the white shape size is lost and arrows are not visible. Please fix.

Reply: The Figure Figure 1 had a transparent background that was shown as black in the PONE submission systems. We now set the background explicitly to white.

Reviewer Point P 1.5 — Line 248, 256, perhaps other lines - starts with a period, please remove.

Reply: This only happens for some unknown reason in the version with track changes for which we use the command line tool *latexdiff*. The referee can be reassured that this does not happen in the final document.

Reviewer Point P 1.6 — Line 283 spelling/style suggestion: "Our work relates to past work, which systematically measure differentials" → "Our work relates to past work that systematically measures differentials"

Reply: Accepted and appreciated

Reviewer Point P 1.7 — Figure 3: Please upper case the ethnic group names on the X axis

Reply: Corrected

Reviewer Point P 1.8 — Figure 3: Please add percentages above the bars to help with interpretation.

Reply: See below

Reviewer Point P 1.9 — Figure 3: The figure is a bit hard to read since it's unclear how the distributions add up to 100%. I recommend restructuring the graph so that each ethnicity-displacement group's bars are stacked and add up to 100%. For example, there would be one stacked bar for non-displaced Kyrgyz that shows the distribution of educational attainment for this group. The figure can then report in the Notes what percent of each group was displaced.

Reply: We created a new improved composite Figure 3 with 95% confidence interval error bars. Using multiple panes reduces the number of bars shown in one graph such that the height of bars can more easily be read and compared. The conclusions remain unchanged.

Reviewer Point P 1.10 — Line 371 - typo: "Osh city" → "Osh City"

Reply: Corrected

Reviewer Point P 1.11 — Line 393 - typo: "ist" → "is"

Reply: Corrected

Reviewer Point P 1.12 — Fig. 4 (and elsewhere when relevant) - I think it would look a little better if the shorthand names for ethnicities (uzb) were upper-cased with a period at the end to show its an abbreviation (Uzb.).

Reply: Corrected

Reviewer 2

Reviewer Point P 2.1 —

The finding that people with lower education levels flee more often is not novel, as I stated in previous report, several scholars have studied the determinants of displacement and found income, education, and vulnerability to be the main drivers.

The authors should exploit this opportunity to explain what is going on behind that correlation, which I believe they don't sufficiently do yet. To put it briefly, the authors say: "We find that educational advantage towards the other ethnic group in the area was associated with a lower probability to flee." Why is this the case? More educated people are more powerful, have higher incomes, are part of the bureaucracy? Have better-defined property rights? Wouldn't these people be precisely the ones that you want to go away? If not, why not?

The lack of clarity on this points, which would give the paper more bandwidth in terms of audience and policy-implications, stems from my view from several issues that are not addressed.

Reply: We thank referee 2 for these thought-provoking questions and comments. We acknowledge that the manuscript did not sufficiently elaborate on the potential mechanisms behind the correlations. We

worked hard to address this issue and hope that the additions to our theory elaborated in the following provide a clear explanation.

We would like to emphasize that it's not education regardless of ethnicity or place that predicts displacement, it's how a person ranks in the local ethnic education distribution.

Reviewer Point P 2.2 —

1. Forced displacement is often strategic, not just the result of the cross fire (plenty of qualitative and quantitative work has been written, including Abbey Steele, Antonella Bandiera, Ana María Ibañez, some which you cite). What is going on here? People have a choice? Why do the the less educated don't have a choice? Someone is forcing them to leave? They don't own property? The place they inhabit can be appropriated?

Reply: To explain how violence leads to displacement, we actually needed a third mechanism between both nodes in our diagram. Adding the node to the graph, we provide the following potential explanation in the theory section:

> *A strand of the literature has looked at the effects of violent conflict on displacement ($\texttt{violence} \rightarrow \texttt{displacement}$) via mechanism M3 (1). On the one hand, civilians might flee out of fear or as a consequence of minimizing risks of harm. Civilians might also follow more forward-looking motives with resetteling strategically to escape future expected targeting by armed groups (2). On the other hand, civilians might decide to stay and fight, or work in lucrative sectors of the war economy. They might decide to stay in order to fight for some cause, join some armed group for personal protection (3), or for personal gain of wealth, assets and power (4).*

Reviewer Point P 2.3 —

2. The current theory is confusing. The authors state there is direct link between ethnic inequality and displacement, that is not mediated by conflict, and they say "We conjecture that, besides violence, a direct connection (ethnic inequalities → displacement) exists and operates through mechanism M2. On the one hand, members of the discriminated group might be able to access better educational opportunities after fleeing. On the other hand, members of the better educated group might be more ready to flee expecting better opportunities for them at arrival." It is hard to understand why they refer to this as displacement, when it sounds it is just voluntary migration. If you have better prospects and mobility is possible, you migrate. However, if the point is that conflict deteriorates economic opportunities and then you are forced to leave, then the relationship is necessarily mediated by conflict.

Reply: We are very grateful about this push-back on the current theory. Referee 2 is absolutely right that current mechanism M2 cannot be reconciled with a short-term movement during a hot phase of conflict. We clearly do measure some form of involuntary displacement. To make this point again we included the survey question along the summary statistics.

> *The survey elicits displacement status during the episode of fighting with the question: "In June 2010, did you have to leave your place of living?". We can furthermore make a distinction of the form of displacement with the follow-up question: "Where did you stay most of the time during the absence?".*

We now describe the potential mechanism as follows in the theory section:

> *We conjecture that, besides observed violence, a direct connection (`ethnic inequalities` → `displacement`) exists and operates through mechanism M2. We propose two alternatives to guide our empirical work. Firstly, the decision to flee might actually be taken based on a subjective perception of risk. Horizontal inequality measures a reality of social hierarchy between individuals of different ethnicities, which might shape the individual belief of the risk to fall victim to aggresions by members of the other ethnic group regardless of observable violence. Secondly, many forms of unobserved violence might exist such as sexual abuse, psychological aggression and socio-economic discrimination which might not correlate perfectly with observed violence such as fighting. Survey data might not be to able to measure these acts as victims might find it difficult to speak precisely about these issues due to stigmatization.*

Discussing the results of the individual regressions, we added the following explanation:

> *Why could inequalities still lead to displacement even though casualties did not occur in their districts? Firstly, simple answer might suggest that there is unobserved violence not necessarily correlated with casualties such as non-lethal physical or psychological violence, discrimination or sexual abuse. Misinformation about the alleged rape of a young Kyrgyz student by Uzbek men mobilized many Kyrgyz men to engage in violence against Uzbeks (5). Due to the stigmatization of victims, international observers judge the assessment of sexual violence generally difficult in Kyrgyzstan (5). However, journalists documented anecdotal evidence of rape by men against women of the other ethnicity, in particular against Uzbek women (6). One could rationalise the decision to flee taken by many horizontally disadvantaged women as a measure to protect themselves and household dependents from the escalating violence between men of both ethnic groups. Secondly, a subjective assessment of risk in ethnic conflict might not necessarily be based on where fighting occurs, but rather how disadvantaged individuals feel relative to members of the other ethnic group. Individuals might not necessarily observe more violence than the statistician, an individual's position objective position in the social hierarchy might explain a subjective assessment of their risk to be targeted by ethnic armed groups.*

Reviewer Point P 2.4 — Lastly, it is hard to understand what is the role of conflict, partly because of the previous points, but also because you don't look at any heterogenous effects by intensity of conflict, or attempt to control using for example a pre-treatment variable that predicts intensity.

Reply:
This is a great comment. Unfortunately the community section is underpowered such that we do not find any significant hetergoeneities with respect to distance to conflict. However, we now include the individual regressions with full interactions with distance to conflict. The results are interesting and are now as discussed as following:

> *Table 8 includes interactions in the specifications reported in Table 7 with the distance to casualties. A 100% percent decrease in the distance to violence is associated with a*

2.3-2.5ppt increase in the base probability of displacement throughout all specifications. Horizontal advantage alone and in interaction with distance to violence are both significant for Uzbeks. Columns D and E show that an Uzbek more educated than most Krygyz in the district he or she lives in is more likely to stay than an Uzbek with lower education. The positive coefficient of interaction implies that this effect is more pronounced the closer the highly educated Uzbek lives to the center of violence. According to our theory, violence leads to displacement through mechanism `M3`*. The fact that horizontally disadvantaged individuals are signficantly more likely to be displaced if violence occurs in their proxmity might be explained through two potential mechanism. Firstly, educational advantage might entail superior information about the conflict. The informational advantage might help to navigate public spaces or find protection by law enforcement. Secondly, horizontal advantaged are more likely to speak Uzbek or Kyrgyz as a second language and better networked to communities across ethnic barriers. They might have been in a better position to rely on interethnic networks to find shelter during the conflict (7).*

We would like to emphasize again that we unfortunately have to work with a post-conflict cross-section. We do have other interesting variables mentioned by the referee or in our theoretical explanations (e.g. assets, self-assessment of security etc.), but they were are all measured ex-post. These variables were likely to be impacted by the decision to flee and therefore do not serve as a right hand side variable.

Reviewer Point P 2.5 — I recommend that the authos re-assess the theoretical analysis: it is still not clear why we should expect more educated people to stay (there are many possible reasons, but the ones you briefly mention are not related to conflict or inequality, rather to the existence of potential economic opportunities elsewhere). Although you have made great progress, without a clear theory that links ethnic inequality in education to displacement (not migration!), we are left with some correlations that are useful but do not allow us to derive any significant policy implications.

Reply:

We thank referee 2 for acknowledging the hard work that went into the first revision. We hope that the renewed revision of our theory contributes to an improved understanding of our findings. We believe that our analysis on a relevant case study contributes to an important debate. Given data limitations, we cannot resolve this debate, but we can still make an important contribution by highlighting relevant mechanisms to be studied further in future studies with better baseline data.

References

[1] Ibáñez AM, Moya A. Who stays and who leaves during mass atrocities? Economic aspects of genocides, other mass atrocities, and their prevention. 2016;251.

[2] Steele A. IDP resettlement and collective targeting during civil wars: Evidence from Colombia. Journal of Peace Research. 2018;55(6):810–824. doi:10.1177/0022343318763706.

[3] Kalyvas SN, Kocher MA. How "Free" Is Free Riding in Civil Wars? Violence, Insurgency, and the Collective Action Problem. World Politics. 2007;59(2):177–216.

[4] Verwimp P. An economic profile of peasant perpetrators of genocide: Micro-level evidence from Rwanda. Journal of Development Economics. 2005;77(2):297–323. doi:https://doi.org/10.1016/j.jdeveco.2004.04.005.

[5] Melvin N. Promoting a Stable and Multiethnic Kyrgyzstan: Overcoming the Causes and Legacies of Violence. Open Society Foundations; 2011.

[6] FerganaNews. Кыргызстан: В Оше похищают девушек-узбечек, насилуют, бреют наголо - и отпускают. Фергана. 2010;.

[7] Akhmetshina M. Loving Your Neighbor as Your Self-Identity: Women's Leading Role in the Interethnic Sheltering During the Osh Conflict of 2010. Central European University. 2012;.
